# Potential predictors of adoption of the Tobacco Heating System by U.S. adult smokers: An actual use study

**DOI:** 10.12688/f1000research.17606.1

**Published:** 2019-02-24

**Authors:** Steve Roulet, Christelle Chrea, Claudia Kanitscheider, Gerd Kallischnigg, Pierpaolo Magnani, Rolf Weitkunat

**Affiliations:** 1 Science and Innovation, Philip Morris International Management S.A., Lausanne, 1007, Switzerland; 2 Science and Innovation, Philip Morris Products S.A., Neuchatel, 2000, Switzerland; 3 Kantar Health GmbH, Munich, Germany; 4 ARGUS Statistics and Information Systems in Environment and Public Health GmbH, Berlin, Germany

**Keywords:** Harm Reduction, Heat-Not-Burn, Modified Risk Tobacco Product, Actual Use, Product Adoption

## Abstract

**Background: **This was a pre-market actual use study with the Tobacco Heating System (THS), a candidate modified risk tobacco product, conducted with adult smokers in eight cities in the United States. The main goal of the study was to describe THS adoption in a real-world setting. The aim of this analysis was to identify potential predictors for adoption of THS using stepwise logistic regression method.

**Methods: **This actual use study was an observational study assessing self-reported stick-by-stick consumption of the THS product compared with the use of commercial cigarettes over six weeks. The study aimed at replicating the usage of THS in real-world conditions with participants being able to consume cigarettes, THS, and any other nicotine-containing products (e.g., e-cigarettes, cigars, etc.) *ad libitum*.

**Results: **14.6% of participants adopted THS, which comprised 70% or more of their total tobacco consumption by the end of the observational period (in Week 6). The main predictors of adoption were the liking of the smell, taste, aftertaste, and ease of use of THS. The proportion of adoption was higher in participants aged 44 years and older and in Hispanic or Latino adult smokers. Additionally, adoption of THS was more likely in participants who had never attempted to quit smoking and in participants who smoked up to 10 cigarettes per day. Finally, the adoption of THS was higher in participants who consumed both regular and menthol THS compared with those who consumed only one THS variant.

**Conclusions:** The main predictors of THS adoption were positive sensory assessment and the ease of use. Socio-demographic characteristics and smoking habits appeared much less important. Post-marketing studies will provide further insights on the impact of the THS at the individual and the overall population level.

## Abbreviations

AIC: Akaike Information Criterion; CDC: U.S. Centers for Disease Control and Prevention; CRF: case report form; FDA: U.S. Food and Drug Administration; MRTP: modified risk tobacco product; THS: tobacco heating system

## Introduction

Cigarette smoking causes pulmonary, cardiovascular, and other serious diseases and is responsible for the largest number of preventable deaths in the United States (U.S.) ^1, 2^. It is widely known that the best way to avoid these risks is to never start smoking. For smokers, the best way to reduce the risks and adverse health consequences of smoking is to quit ^3^. However, as smoking is addictive, smoking cessation has proven difficult to achieve. Despite a decline in the smoking prevalence in the U.S. from 21% to 16% over the last decade, an estimated 40 million people in the U.S. smoked cigarettes in 2015, with around 30% of them smoking menthol cigarettes ^4^.

The U.S. Food and Drug Administration (FDA) and other international health authorities have recognized that in order to more rapidly reduce the burden of death and disease from tobacco use, current tobacco control measures should be enriched and complemented by tobacco harm reduction strategies ^1, 5, 6^. The latter focus on pragmatic goals that aim to provide smokers who do not want to stop nicotine use alternative, noncombustible tobacco and nicotine-containing products or nicotine delivery systems that eliminate exposure to smoked tobacco and thus substantially reduce harm compared with smoking combustible products ^7– 14^.

In the U.S., this has given rise to a regulatory framework for manufacturers to market modified risk tobacco products (MRTP), defined as “any tobacco product that is sold or distributed for use to reduce harm or the risk of tobacco-related disease associated with commercially marketed tobacco products” ^15^.

MRTPs aim to avoid imposing increased risks of chronic disease, morbidity, and mortality at levels caused by smoking cigarettes on their users, and their risk profile is an essential factor in estimating the public health effects of these products ^16^. One approach to harm reduction involves e-cigarettes, which various authorities (Public Health England, 2018; Royal College of Physicians, 2016; U.S. Department of Health and Human Services, 2014) have concluded are likely to be substantially safer than cigarettes. A more recent approach consists of products that heat tobacco rather than burning it, thus producing far lower quantities of harmful and potentially harmful constituents (HPHC) than are found in cigarette smoke ^13^. While it has been acknowledged that more research on the relative risk of heated tobacco products compared with that of combustible tobacco is needed, the available evidence suggests that heated tobacco products may be considerably less harmful than cigarettes ^17, 18^. Currently, the most widely-available heat not burn product is the Tobacco Heating System (THS) developed by Philip Morris International (PMI), sold under the *IQOS* ^®^ brand name. *IQOS* was launched in 2014 in Italy and Japan and is currently available in more than 30 countries. In May 2017, PMI MRTP Applications for *IQOS* with three THS variants were filed for scientific review by the FDA in the U.S. ^19^.

THS uses a precisely controlled heating system into which the THS Tobacco Stick is inserted to generate an aerosol without combusting tobacco. The device heats tobacco to significantly lower temperatures (no more than 350°C) than cigarettes, thereby significantly reducing or eliminating HPHCs from the inhaled aerosol compared with cigarette smoke. The substantial reduction in toxic emission and subsequent body exposure have been established by the THS manufacturer (PMI) and competitors ^20– 35^. Though a few studies have brought contradictory evidence ^36, 37^, the weight of evidence produced by independent studies, including FDA laboratory tests, confirms PMI’s findings on the substantial reduction of major carcinogens ^17, 29, 38– 42^. While prevalence data are still sparse, evidence from Japan, where *IQOS* was first launched, suggest a steady increase in awareness and use of *IQOS* between 2015 and 2017 ^43, 44^. Analysis of predictors of *IQOS* current use (use in the previous 30 days) in 2017 showed that current Japanese smokers with intention to quit had higher odds to use *IQOS* than that of those with no intention to quit (13.3 vs. 6.7), while women aged 60 years or more showed significantly lower odds than reference categories. Ever-use of e-cigarettes was associated with greater odds of using *IQOS*. These findings suggest that the large majority of *IQOS* users in Japan switched from cigarettes to *IQOS* and that there is minimal uptake from nonsmokers. However, they provide limited information on how *IQOS* would impact public health in countries other than Japan. More specifically, in the context of an MRTP application, the FDA recommends assessment of the public health impact of candidate MRTPs under close to real-world conditions to understand how U.S. adult consumers actually use the product ^45^, thus requiring actual use evidence for a product which is not yet commercialized in the U.S. The U.S. Institute of Medicine recommended studies that provide real-world evidence, including *ad libitum* use of MRTPs alone and in combination with cigarettes ^7^.

Although real-world evidence is generally gathered from observational studies in a post-market setting, as with over-the-counter drugs, where consumers are provided with the product together with labeled directions for use ^46– 48^, most of the actual use data that have been collected on potential MRTPs have been done in an artificial setting where the MRTP is provided for free, as opposed to what happens for other commercialized tobacco products in real-life conditions ^49– 51^.

The present study reports the findings of a pre-market actual use study performed in the context of *IQOS* MRTP application to the FDA ^19^. The goal of the study was to measure change of use patterns in U.S. adult daily cigarette smokers and to assess THS product acceptance.

To mimic real-life situations as closely as possible, adult daily smokers had access to THS regular and menthol flavor products and were free to consume cigarettes, THS, and any other nicotine-containing products *ad libitum*.

The present analysis aims at identifying the potential predictors (i.e., socio-demographics, smoking habits, sensory assessment, and ease of use) of THS adoption in adult cigarette smokers. The effect of THS product flavor (i.e., regular or menthol) was also investigated.

## Methods

### Study design

**Figure 1.**
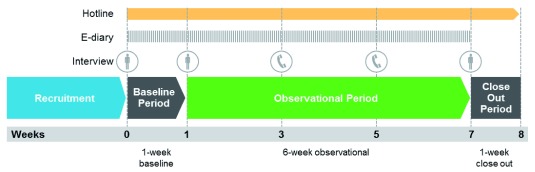
Scheme of study events. * During the baseline and the observational periods, participants recorded their stick-by-stick consumption of cigarettes and/or THS into an electronic diary (e-diary). Participants were able to call the toll-free telephone hotline to raise queries related to the study, resolve issues related to the e-diary or THS, and report product quality complaints and adverse health events associated with the use of THS.

The actual use study consisted of one-week baseline period, a six-week observational period, and a one-week close-out period (see Figure 1) ^19^. During the baseline period, participants recorded their regular cigarette consumption. During the subsequent observational period, participants recorded their consumption of both cigarettes and THS. Throughout the entire observational period, all participants were free to consume cigarettes, THS, and any other nicotine-containing products ad libitum. The observational period served to assess the development of THS use patterns. A close-out period was implemented for safety surveillance.

### Setting

The study was conducted between 21 September 2015 and 7 January 2016 in eight cities located across the U.S. (Asheville, NC; Charlotte, NC; Denver, CO; Detroit, MI; Las Vegas, NV; Miami, FL; Oklahoma City, OK; Tampa, FL). All study materials were reviewed and approved on 28 August 2015 by Sterling Institutional Review Board (ID: 5149-001) before actual study implementation. This study was performed in accordance with Good Epidemiological Practice.

### Participants

Study participants were recruited from the C&C Market Research databases. C&C’s databases consist of approximately 400,000 individuals nationwide who are recruited to join the site database via mall intercept, word of mouth, or by visiting the C&C Market Research website. The sampling was designed using quotas in terms of sex (male (56%); female (44%)), age (18–24 years old (34%); 25–44 years old (34%); 45+ years old (32%)), race (white (70%); black or African American (30%)), and income (low (48%); moderate/high (52%)) ^1^.

Based on information available for each person (e.g., age, gender, smoker/nonsmoker, etc.) within the database, individuals employed by C&C Market Research randomly contacted potential study participants via telephone. No specific method or particular order was utilized for the selection of study participants beyond ensuring that the quotas were met. Individuals who met the following inclusion criteria were eligible for the study: (a) 18 years of age or above according to the minimum legal age), (b) currently living in the U.S., (c) current daily smokers of regular and/or menthol cigarettes with no intention of quitting within the next 30 days, (d) interest in participating in an eight-week study and providing informed consent. The following individuals were excluded from the study: (a) women who, based on self-report, were either pregnant, breastfeeding, or of childbearing potential and not using adequate means of contraception and (b) individuals who had started smoking within the last 30 days. Eligible individuals were then invited to a study site, where they were rescreened for eligibility based on their ID document for proof of age and were asked their intention to use THS based on their reading of a multipage information brochure on THS ( *Extended data* ^52^). Only participants with a positive intention (i.e., “somewhat likely”, “very likely”, “definitely” using a bipolar six-point scale ranging from “definitely not” to “definitely”) were enrolled in the study.

Sample size calculation was based on a precision-based approach (accuracy in parameter estimation) based on predetermined tightness of the confidence intervals. Given a precision of ± 5% for 95% confidence intervals of prevalence estimates and assuming a proportion of 50% of participants passing a consumption threshold of 100 THS products and 40% attrition, the study aimed to recruit 1,300 participants.

### Products

The THS is made up of three distinct components ^20^: (1) a Tobacco Stick, specifically designed for use at low temperatures and containing specially processed crimped tobacco, (2) a holder for the Tobacco Stick that electronically heats the tobacco and controls the temperature, and (3) a charger for recharging the holder after each use. THS was provided by PMI. Products available to participants during the observational period had a neutral design with study identification elements to ensure confidentiality of the THS material, given the pre-market nature of the actual use study. U.S. Surgeon General’s warnings were present on each THS pack in a rotating fashion.

### Data collection and measurements

At enrollment in the study, participants completed an informed consent form and were interviewed in person by trained staff from the C&C Market Research study site in order to provide information on the purpose and goal of the study and instructions on how to use an electronic diary to report tobacco consumption. Questionnaires were also administered to collect demographic information, such as sex, age, race, ethnicity, education, occupation, and income as well as information on smoking habits, including the average number of cigarettes smoked per day, type of cigarette (menthol, regular), current usage of e-cigarettes, current usage of nicotine replacement therapy products, attempts to quit smoking, and the likelihood to use THS regularly as well as the reasons for this.

During the one-week baseline period, participants were requested to make an entry into an electronic diary every time they consumed a cigarette. Upon completion of the one-week baseline period, participants returned to the site to receive THS and choose between THS regular, menthol, or a combination of the two products, according to their taste preference. Participants were provided with a maximum of 100 THS products at the start of the observational period. This supply ensured that all participants had access to THS on the initial days of participation in the observational period. During the remaining study period, participants could request additional THS products. Excessive ordering of additional THS was prevented by fixing an individual maximum number, based on self-reported cigarette consumption assessed at enrollment and then applying an “inflation factor” of three to allow for potential increase of use of THS.

During the six-week observational period, participants were requested to make an entry into the electronic diary every time they consumed a THS or a cigarette. If no entries were made until a predefined time point per day, the e-diary sent an acoustic signal and displayed a reminder to record consumption. E-diary data were transferred automatically to a central database each night. In addition, participants were interviewed every two weeks to assess the taste, smell, aftertaste, and ease of use of THS (telephone interviews at Weeks 3 and 5 and personal interview at Week 7). Taste, smell, and aftertaste were assessed using a seven-point Likert scale ranging from one to seven, where one represented “I don’t like it at all”, and seven represented “I like it very much”. Similarly, ease of use was measured using a seven-point Likert scale ranging from one to seven, where one was “not easy to use at all”, and seven was “very easy to use”.

Participants were able to call the toll-free telephone hotline to raise queries related to the study, resolve issues related to the e-diary or THS, and report product quality complaints and adverse health events associated with the use of THS. At the end of the observational period, participants were asked to return all study materials.

Study participation was voluntary, and participants were free to withdraw at any time. Compensation in the study was based on participants’ level of participation and on compliance with the study procedures (maximum of $440) and paid via check at the end of the study.

### Variables

The main outcome measure was self-reported consumption of cigarettes and THS during the observational period. This measure was used to derive a variable describing the percentage of *THS use* on a weekly basis by dividing the number of THS products by the number of total tobacco products used (THS products plus cigarettes). In order to facilitate meaningful description and interpretation of THS use patterns and future comparison across various studies ^53^, this product use variable was then trichotomized into the following predefined symmetrical usage categories: (1) *THS use* (≥ 70% of total tobacco product used being THS[70–100]% THS), (2) *combined use* (> 30% to < 70% of total tobacco product used being THS ]30–70[% THS), and (3) *cigarette use* (≤ 30% of total tobacco product used being THS [0–30]% THS). In addition, “Adoption of THS” at Week 6 was defined as ≥ 70% of THS products in a participant’s combined consumption of tobacco products during Week 6.

The following variables were evaluated as potential predictors of THS adoption ( Table 1):

**Table 1.** Demographic characteristics and potential predictors by adoption of THS at the end of the observational period.

		Total ^1^	Adoption of THS	No adoption of THS	*p*-value for Chi-square
All participants		965 (100%)	141 (14.6%)	824 (85.4%)	.
Demographics ^2^					
Sex	Male	474 (49.1%)	81 (17.1%)	393 (82.9%)	0.0323
	Female	491 (50.9%)	60 (12.2%)	431 (87.8%)	
Age in categories	18 to 24 years	223 (23.1%)	24 (10.8%)	199 (89.2%)	0.1671
	25 to 44 years	363 (37.6%)	59 (16.3%)	304 (83.7%)	
	Above 44 years	379 (39.3%)	58 (15.3%)	321 (84.7%)	
Persons in household in categories	1 person	216 (22.4%)	38 (17.6%)	178 (82.4%)	0.1591
	> 1 persons	749 (77.6%)	103 (13.8%)	646 (86.2%)	
Children in household in categories	None	615 (63.9%)	96 (15.6%)	519 (84.4%)	0.2586
	1 or more children	348 (36.1%)	45 (12.9%)	303 (87.1%)	
Marital status	No relationship	729 (75.5%)	114 (15.6%)	615 (84.4%)	0.1126
	Relationship	236 (24.5%)	27 (11.4%)	209 (88.6%)	
Occupational status	At work	597 (61.9%)	87 (14.6%)	510 (85.4%)	0.9520
	Not at work	367 (38.1%)	54 (14.7%)	313 (85.3%)	
Educational attainment	Low and moderate	452 (46.9%)	72 (15.9%)	380 (84.1%)	0.2822
	High	512 (53.1%)	69 (13.5%)	443 (86.5%)	
Income levels	Low	334 (36.1%)	54 (16.2%)	280 (83.8%)	0.2424
	Moderate	413 (44.7%)	62 (15.0%)	351 (85.0%)	
	High	177 (19.2%)	19 (10.7%)	158 (89.3%)	
Socio-economic status	Low and moderate	339 (36.7%)	54 (15.9%)	285 (84.1%)	0.3934
	High	584 (63.3%)	81 (13.9%)	503 (86.1%)	
Race	White	653 (67.8%)	88 (13.5%)	565 (86.5%)	0.1376
	Black or African American / Other	310 (32.2%)	53 (17.1%)	257 (82.9%)	
Ethnicity	Hispanic or Latino	115 (11.9%)	27 (23.5%)	88 (76.5%)	0.0041
	Not Hispanic or Latino	850 (88.1%)	114 (13.4%)	736 (86.6%)	
Study location	Asheville	119 (12.3%)	11 (9.2%)	108 (90.8%)	0.1194
	Charlotte	109 (11.3%)	10 (9.2%)	99 (90.8%)	
	Denver	134 (13.9%)	21 (15.7%)	113 (84.3%)	
	Detroit	121 (12.5%)	14 (11.6%)	107 (88.4%)	
	Las Vegas	121 (12.5%)	22 (18.2%)	99 (81.8%)	
	Miami	124 (12.8%)	25 (20.2%)	99 (79.8%)	
	Oklahoma City	111 (11.5%)	16 (14.4%)	95 (85.6%)	
	Tampa	126 (13.1%)	22 (17.5%)	104 (82.5%)	
Smoking behavior					
Average number of cigarettes per day in categories	1–10 cigarettes	405 (42.0%)	72 (17.8%)	333 (82.2%)	0.0318
	11–20 cigarettes	439 (45.5%)	58 (13.2%)	381 (86.8%)	
	≥ 21 cigarettes	121 (12.5%)	11 (9.1%)	110 (90.9%)	
Usage of e-cigarettes	No	913 (94.6%)	129 (14.1%)	784 (85.9%)	0.0756
	Yes	52 (5.4%)	12 (23.1%)	40 (76.9%)	
Intention to quit smoking within the next 6 months	No and don't know	929 (96.3%)	134 (14.4%)	795 (85.6%)	0.4027
	Yes	36 (3.7%)	7 (19.4%)	29 (80.6%)	
Last attempt to quit smoking	Some time in the past	391 (40.5%)	43 (11.0%)	348 (89.0%)	0.0087
	Never	574 (59.5%)	98 (17.1%)	476 (82.9%)	
THS Tobacco Sticks type ordered	Only regular THS Tobacco Sticks	365 (37.8%)	43 (11.8%)	322 (88.2%)	0.0069
	Only menthol THS Tobacco Sticks	424 (43.9%)	59 (13.9%)	365 (86.1%)	
	Both THS Tobacco Sticks types	172 (17.8%)	39 (22.7%)	133 (77.3%)	
	THS Tobacco Sticks consumption type not available	4 (0.4%)	0	4 (100%)	
Product assessment					
Sensory assessments (taste, smell, aftertaste) ^3^	First quartile (< 2.0)	225 (24.0%)	13 (5.8%)	212 (94.2%)	< .0001
	Second quartile (2.0 to < 3.5)	288 (30.7%)	27 (9.4%)	261 (90.6%)	
	Third quartile (3.5 to < 5.0)	209 (22.3%)	35 (16.7%)	174 (83.3%)	
	Fourth quartile (≥ 5.0)	215 (22.9%)	63 (29.3%)	152 (70.7%)	
Ease of use ^4^ assessment	Not easy to use (1,2,3)	301 (32.1%)	18 (6.0%)	283 (94.0%)	< .0001
	Quite easy to use (4,5)	276 (29.5%)	33 (12.0%)	243 (88.0%)	
	Easy to use (6,7)	360 (38.4%)	87 (24.2%)	273 (75.8%)	

^1^ n = 965, excluding three participants without any reported Tobacco Stick or cigarette use within Week 6. Only nonmissing data are shown in the table.

^2 ^Categories recorded in the case report form (CRF) were condensed in order to reduce the number of estimators and balance the number of subjects per category: Persons in household in categories: 1 person, > 1 person

Children in household in categories: None; 1 or more children. Information on children in household was missing for two participants.

Marital status: Relationship (CRF categories: Living with someone / Married), No relationship (CRF categories: Never married / Legally separated / Divorced / Widowed)

Occupational status: At work (CRF category: working now), Not at work (CRF categories: Only temporarily laid off, sick leave or maternity leave / Looking for work, unemployed / Retired /Disabled, permanently or temporarily / Homemaker, keep housing / Student / Other). Information on occupational status was missing for one participant.

Educational attainment: Low (CRF category: less than high school diploma) / moderate (CRF category: high school diploma), High (CRF categories: some university training or university degree). Information on educational attainment was missing for one participant.

Income levels: Low (CRF categories: Less than $30,000), moderate (CRF categories: $30,000 to less than $60,000), High (CRF categories: $60,000 and more). Information on income level was missing for 41 participants.

Socio-economic status is derived as a combination of income levels and educational attainment: Low (low income and low education), Moderate (low income and moderate education, low income and high education, moderate income and low education, and high income and low education), and High (moderate income and moderate education, moderate income and high education, high income and moderate education, and high income and high education). Information on socio-economic status was missing for 42 participants.

Race: White, Black or African American/Other (CRF categories: American Indian or Alaska Native, Asian, Native Hawaiian or Other Pacific Islander). Information on race was missing for two participants.

Last attempt to quit smoking: Some time in the past (CRF categories: less than 6 months ago, more than 6 months ago), Never.

^3^ The taste, smell, and aftertaste of the product were assessed using a seven-point scale ranging from 1 = “I don’t like it at all” to 7 = “I like it very much”. For the scale assessments, Cronbach’s alpha was calculated as measure of internal consistency among the scales. Because of an alpha of 0.89 (above the threshold value of 0.8), a combined construct of sensory acceptance was calculated using the mean scale assessments over taste, smell, and aftertaste. Four categories were created based on the quartiles of the distribution of these mean scale assessments. Information on sensory assessment was missing for 28 participants.

^4^ Ease of use of the product was assessed using a seven-point scale ranging from 1 = “not easy to use at all” to 7 = “very easy to use”. Information on ease of use was missing for 28 participants.

*Demographics*. From the demographic collection at enrollment, the following variables were derived: sex, age (18–24 years, 25–44 years, above 44 years), race (white, black or African American/Other), ethnicity (Hispanic or Latino, not Hispanic or Latino), income (low, moderate, high), number of persons (1 person, > 1 person) and children (none, 1 or more children) in household, marital status (no relationship, relationship), occupational status (at work, not at work), educational attainment (low/moderate, high), socio-economic status (low/moderate, high). In addition, study site location (eight cities) was also considered as a potential demographic predictor.

*Smoking behavior.* From the smoking habits questionnaire at enrollment, the following variables were derived: average number of cigarettes per day (1–10 cigarettes, 11–20 cigarettes, ≥ 21 cigarettes), usage of e-cigarettes (yes, no), intention to quit smoking within the next six months (no or don’t know, yes), last attempt to quit smoking (some time in the past, never). In addition, the type of THS products ordered through the study observational period was also considered as a predictor of THS adoption (only regular, only menthol, both types).

*Product assessment.* Taste, smell, and aftertaste assessment collected at the end of the study (Week 7) were aggregated to quantify sensory assessment into four quartiles. Ease of use assessment was aggregated into three categories (not easy to use, quite easy to use, easy to use).

### Analysis

The study population for analysis included all participants who (1) fulfilled all eligibility criteria, (2) had at least one documented consumption of a cigarette during the baseline period, and (3) had at least one documented consumption of a THS product during the observational period.

Potential predictors of THS adoption underwent bivariate screening using the Chi-squared test (see Table 1). Predictors with a *p*-value < 0.2 were subsequently subjected to stepwise logistic regression, with sex, age, and THS product types ordered being forced-in variables. Backward selection was applied to identify the final model, with *p* < 0.05 as the selection threshold to retain variables. The resulting model was compared with that identified by forward selection using the same variables. In case of a difference between the models, the better model based on the Akaike Information Criterion (AIC) was chosen ^54^.

Additionally, the process was repeated using two-way interaction terms between THS product types ordered and each independent variable with *p*-value < 0.2 from the bivariate screening with simple logistic regressions. The two resulting multiple logistic regression models with and without interaction terms were compared using the AIC.

Analysis was conducted using SAS, version 9.4 (SAS Institute Inc. Cary, NC, USA). All analyses were descriptive and exploratory. No imputation of missing data was applied. Percentages were calculated as proportion of each category based on all nonmissing values.

## Results

### Study participants

Out of the database managed by C&C Market Research, 8,858 members were contacted via telephone. Of these, 1,860 refused to continue the telephone conversation, 5,630 did not meet the eligibility criteria, and the remaining 1,368 were invited to the closest study site and rescreened against inclusion/exclusion criteria to verify eligibility. Of the 1,336 participants who were enrolled into the study, 1,106 participants self-reported at least one cigarette during the baseline period and at least one THS product during the observational period. At the end of the observational period (Week 6), 968 participants had reported data in e-diaries. Of these, three participants reported use of zero THS products or cigarettes. Thus, the analysis population consisted of 965 participants.

The proportion of male participants (49%) in the analysis population was very similar to the proportion of female participants (51%). More than 75% of the participants were 25+ years old, about two thirds (68%) were white, and slightly more than half (56%) had a yearly household income below $45,000 ( Table 1).

### THS product types

Of the analysis population (965 participants), 424 participants (43.9%) ordered only menthol THS products, 365 participants (37.8%) ordered only regular THS products, and 172 participants (17.8%) ordered both types.

### Usage patterns of tobacco products

The proportion of participants with *THS use* decreased between Week 1 (19.4%) and Week 6 (14.6%). Usage patterns of THS products were relatively stable in Weeks 4, 5, and 6 of the observational period.

The proportion of participants with *combined use* (> 30% and < 70% THS) decreased from 41.5% at Week 1 to 22.4% at Week 6, while the proportion of participants with *cigarette use* (≤ 30% THS) increased from 39.0% at Week 1 to 62.7% at Week 6.

The number of tobacco products (THS products and cigarettes) consumed per day during the observational period was lower than the number of cigarettes consumed per day during the baseline period across all participant groups at Week 6. The mean (± standard deviation) number of tobacco products decreased from 9.0 ± 5.89 to 8.1 ± 5.37 in participants with *THS use*, from 9.3 ± 6.34 to 8.9 ± 6.21 in participants with *combined use*, and from 10.9 ± 7.69 to 9.9 ± 6.75 in participants with *cigarette use* ( Table 2).

**Table 2.** Number of THS sticks and/or cigarettes reported per day in different main product use categories ^1^

	*THS use* at Week 6 (n=141)		*Combined use* at Week 6 (n=217)		*Cigarette use* at Week 6 (n=607)	
	Mean	SD	Mean	SD	Mean	SD
*During baseline period*						
Number of cigarettes	9.0	5.89	9.3	6.34	10.9	7.69
*During observational period*						
Number of tobacco products (THS products and cigarettes)	8.1	5.37	8.9	6.21	9.9	6.75
Number of cigarettes	1.4	1.57	4.8	3.72	8.3	6.32
Number of THS products	6.7	4.82	4.1	3.06	1.7	1.99

^1^ Definitions: *THS use*: ≥ 70% of total tobacco product used being THS, (2) *combined use:* > 30% to < 70% of total tobacco product used being THS, and (3) *cigarette use:* ≤ 30% of total tobacco product used being THS.

### Potential predictors of adoption of THS

At the end of the observational period (Week 6), 14.6% of the analysis population had adopted *THS use* ( Table 1). The proportion of participants adopting THS was higher in males (17.1% vs. 12.2%), in participants aged more than 25 years (25 to 44 years: 16.3%, above 44 years: 15.3% vs. 18 to 24 years: 10.8%), in one person households (17.6% vs. 13.8%), in participants with no relationship (15.6% vs. 11.4%), in black or African Americans (17.1% vs. 13.5%), and in Hispanic or Latino participants (23.5% vs. 13.4%).

With regard to smoking habits, the proportion of participants adopting THS was higher in participants smoking from one to 10 cigarettes per day (17.8% vs. 11 to 20 cigarettes per day: 13.2% and ≥ 21 cigarettes per day: 9.1%), e-cigarette users (23.1% vs. 14.1%), and in participants who never attempted to quit smoking (17.1% vs. 11.0%). The proportion of participants who adopted THS was higher in those who ordered both THS products (22.7% vs. 13.9% for menthol only vs. 11.8% for regular only).

The proportion of participants adopting THS was higher in participants who liked the taste, smell, and aftertaste of THS (increasing from 5.8% in the first quartile to 29.3% in the fourth quartile for sensory assessment scores) and in participants who found THS easy to use (increasing from 6.0% in participant who found THS not easy to use to 24.2% in participants who ound THS easy to use).

**Figure 2.**
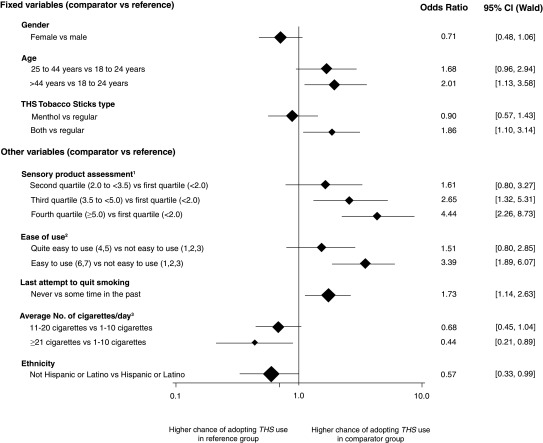
Predictors of adoption of THS at the end of the observational period. The vertical line shows the value where chances of adopting are equal in both the reference and the comparator group. Horizontal lines show the confidence intervals. The size of the diamonds is proportional to the number of participants in the comparator group. ^1^ The taste, smell, and aftertaste of the product were assessed using seven-point scales ranging from 1 = “I don’t like it at all” to 7 = “I like it very much”. For the scale assessments, Cronbach’s alpha was calculated as measure of internal consistency among the scales. Because of an alpha of 0.89 (above the threshold value of 0.8), a combined construct of sensory acceptance was calculated using the mean scale assessments over taste, smell, and aftertaste. Four categories were created based on the quartiles of the distribution of these mean scale assessments. ^2^Ease of use of the product was assessed using a seven-point scale ranging from 1 = “not easy to use at all” to 7 = “very easy to use”. ^3^Average number of cigarettes/day at enrollment.

Stepwise main effects logistic regression analysis resulted in the same model, max-rescaled R-square of 0.1968 and 76.2% of concordant pairs, regardless of the selection method (i.e., forward or backward). The predictors of adoption of THS at the end of the observational period are summarized in Figure 2.

No influence of sex (OR = 0.71 [95% CI: 0.48–1.06]) was found, but adoption of THS was more likely in participants aged more than 44 years (OR = 2.01 [95% CI: 1.13–3.58]) and in participants who ordered both THS product types (OR = 1.86 [95% CI: 1.10–3.14]).

Sensory assessment and ease of use were the main predictors for THS adoption *.* The odds of adopting THS were more than four times higher in participants who liked the smell, taste, and aftertaste of THS (≥ 5.0 points on a seven-point scale) (OR = 4.44 [95% CI: 2.26–8.73]). Similarly, the odds to adopt THS were more than three times higher in participants who found THS easy to use (OR = 3.39 [95% CI 1.89–6.07]).

Participants who had never attempt to quit smoking had a higher chance of adopting THS compared with those who attempted to quit at some time in the past (OR = 1.73 [95% CI 1.14–2.63]).

Participants who smoked on average ≥ 21 cigarettes/day had a lower chance of adopting THS compared with those who smoked on average 1–10 cigarettes/day (OR = 0.44 [95% CI 0.21–0.89]), and the same applied for non-Hispanic or Latino participants compared with Hispanic or Latino participants (OR = 0.57 [95% CI 0.33–0.99]) ( Figure 2). Interaction terms with the consumed THS product type did not improve the overall model fit.

## Discussion

The main goal was to describe THS adoption in a real-world setting and to identify potential predictors for adoption of THS. This actual use study was conducted in U.S. adult daily smokers and included 1,106 participants self-reporting their consumption of cigarettes and/or THS products using an electronic diary. The study was conducted in eight cities spread across the U.S. to recruit a sufficiently large and diverse number of U.S. adult daily smokers. Quota sampling in terms of sex, age, race, and income was applied.

The proportion of participants with *THS use* was stable from Week 4 onwards. By Week 6, almost 15% of the participants had adopted THS, suggesting that THS is a viable alternative to cigarettes for adult smokers. The results do not indicate an increase of tobacco consumption over the observational period. Therefore, even though dual use is likely to happen in the first weeks of THS use, it is unlikely to lead to higher abuse liability and increase exposure to tobacco and nicotine products.

The adoption of THS was higher in participants ordering both THS types compared with participants ordering only regular or only menthol THS, suggesting that the availability of several variants of THS, including menthol, might result in a higher proportion of U.S. adult smokers substituting cigarettes with THS. Similar findings have been reported in studies with electronic cigarettes and noncombustible nicotine products ^55– 58^. Some of these studies also indicated that the use of menthol can facilitate the transition from cigarettes to reduced-risk products (RRP) ^55– 58^. “RRPs” is the term that PMI uses to refer to products that present, are likely to present, or have the potential to present less risk of harm to smokers who switch to these products versus continued smoking.

Participants who liked the smell, taste, and aftertaste of THS and participants who found THS easy to use were more likely to adopt THS, compared with participants who did not like THS smell, taste, and aftertaste or did not find THS easy to use. This finding supports results from previous studies that found that one of the main reasons that people stop using e-cigarettes after trying them is that they do not like the taste ^58– 61^.

Participants smoking 1–10 cigarettes per day were more likely to adopt THS than participants smoking more than 21 cigarettes/day. A similar outcome has been reported for e-cigarettes, as indicated by the prevalence of regular use of e-cigarettes being higher among adult smokers who smoke a lower number of cigarettes per day ^62^.

Participants who had previously attempted to quit smoking were less likely to adopt THS than participants who never attempted to quit smoking in the past. This finding suggests that the availability of THS is unlikely to prevent those willing to quit tobacco from doing so. This is further confirmed by the fact that the intention to quit smoking within the next six months was not associated with THS adoption.

The proportion of THS adoption was higher in participants aged 44 years and older compared with participants aged between 18 and 24 years old. Hispanic or Latino participants had a slightly higher likelihood of adopting THS than not-Hispanic or Latino participants.

Other demographic characteristics, such as sex, household size, educational attainment, income levels, or race, were not associated with THS adoption.

Overall, these findings show that the socio-demographic characteristics of smokers who are more likely to adopt THS tend to differ from what has been recently reported on e-cigarettes, particularly in terms of age, ethnicity, and previous quit attempts ^62^. This suggests that THS may be seen as an acceptable substitute for cigarettes to a different category of smokers than those who are currently using e-cigarettes. This is corroborated by the fact that current e cigarette use was not associated with THS adoption. 

Importantly, the study findings highlight the importance of offering alternatives that are close to cigarettes from a sensory experience for the adoption of RRPs ^58– 61^, with product liking and ease of use being more important predictors for adoption of THS than socio-demographic characteristics and smoking habits.

The key strengths of this actual use study included (1) the high ecological validity due to the near to real-world setting of the study, (2) the broad regional coverage, (3) the large sample size, and (4) the duration of the observational period of six weeks (which is slightly longer than in previous studies of alternative tobacco products ^63, 64^).

Limitations include the fact that due to the study having been conducted in a pre-market setting, the study participants did not pay for the THS products, while they continued to pay for their cigarettes, which may have overestimated the level of THS adoption in this study. Also, the sample was not representative of the U.S. adult smoker population, which should be considered when interpreting the results. Finally, no biochemical verification of tobacco consumption, such as CO monitoring, was used, as the method of data collection relied exclusively on self-reported tobacco consumption. With regard to this point, it should be noted that validation studies have shown that self-reported tobacco consumption behaviors among adults are consistent and reliable ^50, 65^.

Factors that were not measured may have influenced THS adoption (e.g., repeated exposure to product communication, peer-to-peer information sharing, risk perception [the product possibly being perceived as possible risk-reduced], familiarity, and acceptability of alternative tobacco usage behavior, as it may develop once the product has been marketed for some time) ^66^.

In view of the above limitations, post-market studies are needed to provide actual levels of THS adoption and use patterns once THS is commercially marketed in the U.S. More studies are also needed to further understand what the drivers of THS adoption are. Consistent with several theoretical frameworks that have been used to understand the impact of intervention or prevention policies ^67, 68^, research should not only look at factors intrinsic to the users or to the product to explain use behavior but also take into consideration the influence of social (e.g., family background, peer influence) and societal/environmental factors (e.g., media influence, public health policy).

## Conclusions

This actual use study showed that after a six week period of *ad libitum* use of THS provided at no expense, almost 15% of U.S. daily adult smokers substituted cigarettes with THS.

In this context, potential predictors for adoption of THS were a positive opinion of the taste, smell, and aftertaste and the ease of use, while socio-demographic characteristics and smoking habits were less important. Adoption of THS was higher in smokers aged over 44 years and in Hispanic or Latino smokers. For other demographic characteristics, such as sex, household size, educational attainment, income levels, or race, an influence on the adoption of THS was not found. Moreover, the adoption of THS was higher in smokers who used both menthol THS and regular THS products.

The results suggest that the introduction of THS in the U.S. has the potential to result in adoption by adult smokers who would otherwise continue to smoke cigarettes. On the basis of this adoption rate, this could benefit public health by having a positive impact on this particular population of adult smokers ^69^. In particular, the results indicate that the adoption of THS is unlikely to result in an increase of tobacco consumption. Epidemiologic and post-marketing studies can provide further insights on the impact of the THS at the individual and the overall population level.

## Data availability

### Underlying data

Open Science Framework: Potential predictors of adoption of the Tobacco Heating System (THS) by U.S. adult smokers. https://doi.org/10.17605/OSF.IO/SBDXG ^52^.

This project contains the following underlying data files:

- Raw dataset.sas
- Variable Coding Book.pdf

### Extended data

Open Science Framework: Potential predictors of adoption of the Tobacco Heating System (THS) by U.S. adult smokers. https://doi.org/10.17605/OSF.IO/SBDXG ^69^.

This project contains the following extended data files:

- Brochure.pdf

Data are available under the terms of the Creative Commons Zero "No rights reserved" data waiver (CC0 1.0 Public domain dedication).

## Footnotes

^1^Low income (annual household ≤ $44,999); moderate/high income (annual household ≥ $45,000).
