# Supplementary material for: Potential predictors of adoption of the Tobacco Heating System by U.S. adult smokers: An actual use study
Source: F1000Res. 2021 Jul 14;8:214. Originally published 2019 Feb 24. [Version 2] doi: 10.12688/f1000research.17606.2 (PMC6743249; doi:10.12688/f1000research.17606.2)
Supplement: Peer review history

# Peer review: Reviewer response for version 1

**DOI:** 10.5256/f1000research.19251.r52579

**Published:** 2019-09-11

**Authors:** Krishna Prasad, Fabrizio Messina

**Affiliations:** 1 BAT (Investments) Limited, Southampton, UK; 2 Research and Development, British American Tobacco, Southampton, UK

This paper reports the outcome from an ‘actual use’ study designed to determine if users are likely to use the product in a manner that reduces their individual health risks or exposures as compared to using other commercially marketed tobacco products. Actual use studies should allow consumers to interact freely with the product in real world conditions.

 The study design, recruitment, methodology, data collection, analysis and results are well presented. However, some of the conclusions drawn are broad and statistical procedures used are unconventional, leading to interpretation based on variable selection criteria. Given the recruitment limitation of not having recruited non-tobacco users, the conclusions drawn should be limited to THS adoption among current smokers in the US.

**Abstract**

 The objective of the study is stated twice in the background.

 Observational studies by their very nature observe individuals without manipulation or intervention. In this study the participants are asked to use the test products instead of their regular products, therefore, should be referred to as actual use study.

 There is little if any data regarding the use of e-cigarettes, cigars etc. in the body of the paper. Number and type of participants and brief inclusion/exclusion criteria would make the methods section more informative.

 Whilst I understand that it is not for the paper to make policy implications, it would be useful to draw on the adoption of THS by vulnerable groups in conclusions.

**Introduction**

 Generally, the introduction is well presented with relevant references from the literature. Reference to actual use studies with similar tobacco heating system would enhance the articles and provide basis for comparison in discussion.

 Despite acknowledging that most actual use studies for cMRTP have been conducted in artificial setting and provided free, the products were provided free in this study.

**Methods**

 The methods used are clearly presented with reference to sample size calculation, inclusion/exclusion criteria, products, data collection and analysis.

 Rationale for some of the criteria for example, exclusion of individuals who had started smoking with in the last 30 days would have helped.

 Sample Size Calculation

 Based on the reference in the paper (Dhand, N. K., & Khatkar, M. S. (2014)). Calculating the sample size

 n ≥ (1.96/0.05)^2 *0.5 × 0.5 = 384.16 and allowing for an attrition rate of 40%, I arrive at 384.16*(1/(1 - 0.4))= 641.

 In this paper they have recruited 1300 which is almost double as 641.

 Pre-screening of the regressors

 I understand the practice of pre-screening the regressors to understand the relation between the dependent and the independent variables, however, excluding regressors based on an arbitrary rule (p-value <=0.2), it’s rather unconventional.

 According to “Five myths about variable selection” by Georg Heinze & Daniela Dunkler ^1^: “While it is true that regression coefficients are often larger in univariable models than in multivariable ones, also the opposite may occur, if some variables (with all positive effects on the outcome) are negatively correlated. Moreover, univariable prefiltering, sometimes also referred to as “bivariable analysis,” does not add stability to the selection process as it is based on stochastic quantities and can lead to overlooking important adjustment variables needed for control in an etiologic model. Although univariable prefiltering is traceable and easy to do with standard software, one should better completely forget about it as it is neither a prerequisite nor providing any benefits when building multivariable models ( *Sung et al 1996) ^2^.”*

 If the authors have not already run the logistic regression including all the regressors, it is worth re-running to see if we observe different potential predictors.

- The study participants were asked to report the current use of NRT products – despite selecting smokers who had no intention to quit. What was reasons for them using NRT?
- The main outcome measure was self-reported consumption of cigarettes and THS, does not take into account e-cig or NRT use - this would likely influence the number of THS sticks used and therefore inflate the ratio?

**Results**

- Is the decrease in the number of tobacco products (Table 2) significant? Given the large SDs I suggest they are not different.

**Discussion**

- Can you say that THS is a viable alternative if 85% of users rejected the offer even when given the product for free?
- "availability of THS is unlikely to prevent those willing to quit tobacco from doing so". Can you really make this statement from the data provided? 'Previous quit attempts' is different from 'intention to quit'.
- "almost 15% of U.S. daily adult smokers substituted cigarettes with THS". While this is technically true, they didn't substitute completely which may be incorrectly inferred from this conclusion.

Is the work clearly and accurately presented and does it cite the current literature?

Yes

If applicable, is the statistical analysis and its interpretation appropriate?

Partly

Are all the source data underlying the results available to ensure full reproducibility?

Yes

Is the study design appropriate and is the work technically sound?

Yes

Are the conclusions drawn adequately supported by the results?

Yes

Are sufficient details of methods and analysis provided to allow replication by others?

Yes

Reviewer Expertise:

I have several years’ experience in tobacco and nicotine products use behaviour. As part of my current role I look after all human studies from mouth level exposure, puffing topography, consumer risk perception and post market surveillance.

We confirm that we have read this submission and believe that we have an appropriate level of expertise to confirm that it is of an acceptable scientific standard.

## References

[1] : Five myths about variable selection. *Transpl Int* .2017;30(1) : 10.1111/tri.12895 6-10 10.1111/tri.12895 27896874

[2] : Inappropriate use of bivariable analysis to screen risk factors for use in multivariable analysis. *J Clin Epidemiol* .1996;49(8) : 10.1016/0895-4356(96)00025-x 907-16 10.1016/0895-4356(96)00025-x 8699212

# Response

**Published:** 2021-06-20

**Authors:** Christelle Chrea

**Affiliations:** Philip Morris, Switzerland

**Abstract**

 The Abstract has been slightly edited according to the Reviewers’ comment on its structure, with no alteration to the message presented. (i.e. removed repetitious sentences, as well as addition, restructuration and move of sentences to a different part of the abstract for clarity, etc…). As suggested, the number of participants together with the description of the participants (i.e. current adult smokers) is now included in the Abstract. The full description of inclusion/exclusion criteria remains included under Participants subsection of the Methods.

 The Reviewers indicated that because the participants are asked to use the test products instead of their regular products, it should be referred to as an actual use study in the Abstract. We fully agree with this statement and, actually, the manuscript title "Potential predictors of adoption of the Tobacco Heating System by U.S. adult smokers: An actual use study" clearly reflects that it is an actual use study. The terminology of "observational study" is also being used to refer to the fact that, there was no intervention with the study population beyond providing the THS product. Moreover, study participants were not asked to use the THS product during the entire study periods (i.e. baseline and observational periods); they were only requested "to make an entry into an e-diary every time they consumed a cigarette during the 1-week baseline period and a HeatStick or a cigarette during the 6-week observational period of the study. Participants were also requested to indicate on a daily basis using the e-diary whether or not they used other products containing nicotine (i.e. e-cigarettes, nicotine replacement therapy products, and other tobacco products such as cigars, cigarillos and smokeless tobacco products)."

 The Reviewers suggested to further expand our conclusions and draw on the adoption of THS by vulnerable groups. THS is aimed at current adult smokers who would otherwise continue to smoke combustible cigarettes and represent the study population of this actual use study. Therefore, we believe that drawing any conclusion from this study regarding vulnerable groups is beyond the scope of this publication.

 The Reviewer noted that the data about the use of e-cigarettes, cigars and other tobacco products than cigarettes, is not extensively covered in our manuscript. This is because the Tobacco Heating System is aimed at current adult smokers who would otherwise continue to smoke cigarettes and hence the focus of this publication is on THS and cigarettes. Data about e-cigarette use has been captured during the rescreening phase (yes/no) and show that "the proportion of participants adopting THS was higher among e-cigarette adult users (23.1% vs. 14.1%)". Data about e-cigarette use, as well as nicotine replacement therapy products, and other tobacco products such as cigars, cigarillos, and smokeless tobacco products, has also been captured during the baseline and the observational periods. The data show that between the baseline and the end of the observational period, the usage of nicotine replacement therapy products remained stable, while the usage of e-cigarettes increased and the usage of other tobacco products such as cigars, cigarillos and smokeless tobacco products decreased.

**Introduction**

 The Reviewers outlined that reference to actual use studies with a similar tobacco heating system (THS) would enhance the articles and provide basis for comparison in discussion. While we agree with this comment, we are not aware of any other premarket actual use study regarding another heated tobacco product (i.e. beyond the THS).

 Regarding the study settings, the studies referenced in Introduction which collected data on potential MRTPs were performed in an artificial setting because there was an intervention (i.e. participants being instructed to adopt a given behavior), which was not the case for our study. We slightly edited the related sentence to clarify that for these studies, the MRTP was also provided for free, as it was also the case for our pre-market study. This aspect of the study design has been stated as a study limitation in the Discussion section of the manuscript, which states, "Limitations include the fact that due to the study having been conducted in a pre-market setting, the study participants did not pay for the THS products, while they continued to pay for their cigarettes, which may have overestimated the level of THS adoption in this study."

**Methods**

 The Reviewer requested further insights regarding the rationale for some of the inclusion/exclusion criteria, in particular concerning the exclusion of individuals who had started smoking within the last 30 days. This exclusion criteria was applied to ensure that participants involved in the study have been smoking cigarettes for a certain period of time, and thus, have a relatively established product use pattern (of using cigarettes, in this particular case).

 The Reviewers provided a correct sample size calculation (i.e. 641 participants). Additionally to the number of 641, we assumed that we will have 50% "starters" ("starters" are those subjects who used 100 or more HeatSticks during the observational period)  in the sample in addition to the 40% related to the attrition rate, which doubles the calculated number. Thus, we recruited 1300 participants.

 To the Reviewers comments on pre-screening of the regressors, we saw that different authors have favored different variable selection strategies. We have chosen this procedure to have sufficient degree of freedoms to estimate the effects. It seems unlikely that the variables in our dataset with p>0.2 in the bivariate analysis will have an additional influence.

 The Reviewers formulated questions regarding reasons for participants using NRT and whether number of HeatSticks used could have been influenced by use of e-cigarettes and NRT. The main data for this manuscript was the number of cigarettes and the number of HeatSticks used by study participants over a 6-week period. Information related to the use of e-cigarettes, cigars etc. can be found as part of the study report, which is publicly available as part of Philip Morris Products S.A. Modified Risk Tobacco Product (MRTP) Applications and can be accessed through the following link: https://www.fda.gov/tobacco-products/advertising-and-promotion/philip-morris-products-sa-modified-risk-tobacco-product-mrtp-applications. The percentage of NRT use was 0.1% at rescreening, which indicates that it is highly unlikely to have any impact on the number of HeatSticks used.  The percentage of e-cigarette use was 5.3% at rescreening so relatively small, and hence, unlikely to have any significant impact on the number of HeatSticks used. 

**Results**

 The Reviewers commented on the potential significance of the decrease in the number of tobacco products presented in Table 2. Actually, the results are reported in means without any statistical test.

**Discussion**

 The Reviewers asked whether stating that THS is a viable alternative was appropriate with 85% of users rejecting the offer even when given the product for free. We believe that THS is a viable alternative as about 15% of current adult smokers’ participants have replaced 70% or more of their tobacco consumption (cigarettes + THS) by THS. Further analysis has shown that about 6% (n = 968) of adult smokers’ participants completely stopped smoking combustible cigarette (i.e. THS constituted 100% of their tobacco consumption).

 As per the Reviewers comment regarding previous quit attempts and intention to quit, the manuscript has been amended to address your comment as follows: the old text "Participants who had previously attempted to quit smoking were less likely to adopt THS than participants who never attempted to quit smoking in the past. This finding suggests that the availability of THS is unlikely to prevent those willing to quit tobacco from doing so. This is further confirmed by the fact that the intention to quit smoking within the next six months was not associated with THS adoption," was replaced with “Participants who never attempted to quit smoking in the past were more likely to adopt THS than participants who had previously attempted to quit smoking. Intention to quit smoking within the next six months was not associated with THS adoption suggesting that the availability of THS is unlikely to interfere with intention to quit smoking."

 When it comes to cigarettes substituted with THS for study participants, the manuscript has been amended as follows: the old text "Almost 15% of U.S. daily adult smokers substituted cigarettes with THS" was replaced with "almost 15% of U.S. daily adult smokers replaced 70% or more of their tobacco consumption with THS".

# Peer review: Reviewer response for version 1

**DOI:** 10.5256/f1000research.19251.r52578

**Published:** 2019-09-02

**Authors:** Riccardo Polosa

**Affiliations:** 1 Centre for the Prevention and Treatment of Tobacco Addiction (CPCT), Teaching Hospital “Policlinico-V. Emanuele”, University of Catania, Catania, Italy; 2 Center of Excellence for the acceleration of Harm Reduction, University of Catania, Catania, Italy

In this 6-weeks observational study, self-reported stick-by-stick consumption of IQOS was assessed and potential predictors of product adoption identified. Understanding predictors of e-cig/THP adoption is important as it may lead to improved smoking cessation/reduction rates. I have the following comments:

**Major Comments**

 Recruiting method is a potential (and probable) source of bias. As noted by the Authors, market panel members are not a representative sample of the population of US smokers. The participant remuneration was significantly high ($440); this - together with the fact that IQOS and consumables were given for free - would encourage participation and spin study findings. All the above casts doubts on whether the study is really conducted under real-world setting conditions and jeopardize the main goal of the study, which was to describe IQOS adoption in a real-world setting.

 The study should be better reported.

- No report of refusal to participate.
- No report of partial compliance with diary reporting. 
- No report of the number non-compliant with interview schedule.
- No report of the number not turning in study materials (used for measurement, so significant).
- The only report is the number of participants who made no diary entries, and that alone is significant as 28% made no entries. If the authors conducted imputations for missing data (and certainly there were some missing data points!) this should be reported. 

I have concerns about the Analyses.

- E-cig users made up 12 of the 141 adopters, but represented only 5.4% of the population - clearly skewing the results. E-cig use should have been analyzed as a confounder. 
- The combined use categories make little sense to me.
- The effectiveness for Hispanics/Latinos barely reached significance.
- Wide CIs indicate that there are insufficient numbers for subgroup analysis.

Can you clarify which version of the product was provided in this study; as far as I am aware the manufacturer has rolled out the third generation/evolution of IQOS. This is important for the interpretation of study findings (I was told that newer generations perform substantially better than earlier generations).

 Findings are products specific. It would have been interesting to have another comparator (e-cigs?) in the study in order to have a better understanding in terms of predictors of adoption of these new technologies. This should be discussed.

 There’s lack of information about complete abstinence from tobacco cigarettes and this should be provided.

**Minor Comments**

 Introduction, Page 3. *“* *These findings suggest that the large majority of IQOS users in Japan switched from cigarettes to IQOS and that there is minimal uptake from nonsmokers”. *Please qualify these statements with appropriate numbers/percentages (and references).

 The authors state that the study supplied a hotline for information and to collect reports of adverse effects. How many calls did the hotline receive? What adverse effects were reported? This is critical information and should be provided in the paper.

 Study design. Is there a psychological behavioural pharmacological theory/rationale for the chosen length of study periods (i.e. 1 week for baseline period; 6 weeks for observation period)?

 I note that a validated psycho-diagnostic tool was used to measure participants’ intention to quit. Please specify which one.

 More information on the structure and validity of the questionnaires used are need and the questionnaires should be included in the appendix.

 It would have been equally important to evaluate the construct of the intention to switch to low-risk products.

 An important predictor for IQOS adoption could have been the participants own cigarette brand.

 I was sorry to see in the analysis that heavy smokers (21+/day) were less likely to adopt than light smokers. This may reflect high level of inefficiency of (currently marketed) IQOS to adequately reproduce the experience in cigarette smoking. This should be discussed.

Is the work clearly and accurately presented and does it cite the current literature?

Yes

If applicable, is the statistical analysis and its interpretation appropriate?

Partly

Are all the source data underlying the results available to ensure full reproducibility?

No

Is the study design appropriate and is the work technically sound?

Partly

Are the conclusions drawn adequately supported by the results?

Partly

Are sufficient details of methods and analysis provided to allow replication by others?

Partly

Reviewer Expertise:

Tobacco research (including ECIG and THP)

I confirm that I have read this submission and believe that I have an appropriate level of expertise to confirm that it is of an acceptable scientific standard, however I have significant reservations, as outlined above.

# Response

**Published:** 2021-06-20

**Authors:** Christelle Chrea

**Affiliations:** Philip Morris, Switzerland

**Major comments**

 The Reviewer questioned the recruitment method and the extent to which our study should be considered to have been conducted under real-world setting conditions. From our perspective, an Actual Use Study represents the most appropriate study design to approximate real-world conditions in a premarket environment (i.e. in a condition in which the investigational product has not been authorized to be marketed, and therefore, is not commercially available to the public yet).  Therefore, such study attempts to replicate to the maximum possible extent within the boundaries determined by the pre-market conditions and the limits imposed by feasibility.

 With respect to the recruiting method, we would like to note that the rationale for the selection of a non-probabilistic sample was to ensure the feasibility of the study. The sample was large as more than 1,300 participants were enrolled in the study and the results were robust as the study was conducted in eight different sites spread across the U.S.

 We chose these eight geographical locations to: i)  recruit a diverse population of current U.S. adult daily smokers study participants; ii) allow distribution of the product, considering the various state requirements (e.g., retail license, tax stamping by a licensed distributer, pre-notification to fire Marshall); and iii) to ensure a good control over the investigational product. Although results are not generalizable to the U.S. smoking population, they provide a robust description of the patterns of use.

 Concerning the participants remuneration, we believe that participants were reimbursed adequately for their time and effort. The compensation was based on the study duration and the numerous tasks that they were asked to perform, e.g., complete an e-diary on a stick-by-stick frequency, answer to CATI interviews, go to the study site to get additional product, keep used and unused product, etc.  Additionally, compensation in the study was up to $440, depending on the length of time in the study and return of the THS, used and unused *HeatSticks*, and the e-diary device. Assuming a full participation, this means $55 per week. To minimize the impact that reimbursement could have on participants’ tobacco consumption, each participant was reimbursed only after the completion of the study. The amount of the compensation was approved by the Institutional Review Board (Sterling IRB) and it was in line with the consumer research standards in the U.S. Finally, THS and *HeatSticks* were given for free, which is normal practice in consumer research, as stated by the ESOMAR code. We also note that other different features might have discouraged product use compared to a full real-world setting, e.g., lack of repeated communication, absence of a real customer care and presence of other adult users using the product. Having said that, we recognize that some of the points made by the Reviewer are the limitations of the study, which were inherent to the pre-market setting, and have been stated as such in the manuscript.

 The Reviewer recommended to further report the overall disposition of subjects. The aim of this analysis was to assess the potential predictors of adoption of the Tobacco Heating System (THS) by current U.S. adult smokers after six weeks of THS product use. The data from this last week of the observational period (i.e. week 6) was therefore appropriate. In terms of the overall disposition of subjects, at pre-screening, 8,858 subjects, spread over eight sites (ranging from 599 in Denver to 2,050 in Detroit), were contacted via telephone. Of these, 21% initially refused to continue the telephone conversation. A certain proportion of the remaining subjects did not fulfill pre-specified criteria. The most common of these unfulfilled criteria were as follows: (i) participation in a consumer or clinical research study in the past three months (32%), (ii) being a non-daily adult smoker (18%), (iii) not willing to participate in a consumer research study that could last up to eight weeks (10%), (iv) started smoking regularly within the past 30 days (10%), (v) and demographic criteria above quota (10%). After this pre-screening phase, subjects were invited to one of the eight sites. At the eight sites 1,368 subjects were screened in person. Of the 1,368 screened subjects, 1,336 participants were enrolled, while 32 participants were not enrolled because of violation of inclusion or exclusion criteria. The number of participants screened was overall similar for each of the eight sites (ranging from 162 in Oklahoma City to 179 in Miami). The Full Analysis Set (FAS) comprised 1,106 participants, of which 119 prematurely discontinued and 987 completed the study. Overall, 230 subjects enrolled were not included in the FAS because they did not self-report in the e-diary “at least one documented consumption of a cigarette during the one-week baseline period” or “at least one documented consumption of a *HeatStick* during the six-week observational period.” Moreover, no imputation of missing information was applied as part of the study. Those details can be found as part of the full study report, which is publicly available as part of Philip Morris Products S.A. Modified Risk Tobacco Product (MRTP) Applications and can be accessed through the following link: https://www.fda.gov/tobacco-products/advertising-and-promotion/philip-morris-products-sa-modified-risk-tobacco-product-mrtp-applications. 

 The Reviewer formulated concerns about the analyses. The number of e-cigarette adult users is too small from our perspective to put into the logistic model (i.e. as a potential confounder) and the estimation of an effect would lead to wide Confidence Intervals (CIs). Regarding the effectiveness for Hispanics/Latinos, this is a true statement coming from the analysis. This means that this subgroup is no more likely than other subgroups to use THS; the reporting of the results was performed in line with the multivariate analysis, presented in Figure 2. The Reviewer outlined that wide CIs indicate that there are insufficient numbers for subgroup analysis. This is indeed correct as the planning of the sample size was not done for subgroup analysis.

 Considering the potential inclusion of another comparator such as e-cigarettes, this suggestion from the Reviewer is interesting and we believe it merits potential exploration in studies designed and executed with a different purpose than a Modified Risk Tobacco Product Application. We have noticed that the Reviewer has designed such a study to compare changes in cigarette consumption and adoption rates among smokers randomized to either IQOS or e-cigarettes: https://www.ncbi.nlm.nih.gov/pmc/articles/PMC6962654/.

 Regarding information about complete switching from cigarettes (i.e. 100% THS use), indeed, this information was not intended to be part of the manuscript, however, it has been provided during the Tobacco Products Scientific Advisory Committee review of Philip Morris Products S.A. Modified Risk Tobacco Product (MRTP) Applications and can be accessed through the following link: https://www.fda.gov/media/110734/download. The percentage of 100% THS use (i.e. completely stopped using combustible cigarettes) was 6% at Week 6.

 Under the Variables sub-section of the Methods, we provide the definition of the combined use category. To the Reviewer’s comment about this category, we would like to outline that we have defined the product use categories with the objective of having a symmetrical classification, which allowed for practical and meaningful operationalized categorization, description and analysis. We have provided the raw data to allow scientists to create further categories.

 Regarding the version of the product provided for the study, the Products sub-section of the Methods has been edited to indicate that the investigation product as part of this actual use study was the Tobacco Heating System Version 2.2. Thus, the product used in this study was an earlier version of the currently commercialized THS product.  

**Minor Comments**

 In the Introduction, the Reviewer suggested to qualify our statement “These findings suggest that the large majority of IQOS users in Japan switched from cigarettes to IQOS and that there is minimal uptake from nonsmokers” with numbers/percentages (and references). The findings come from several studies that are cited earlier in the text including their respective references (Tabuchi, 2017 and Tabuchi, 2016). Therefore, stating one particular percentage does not seem adequate as the statement is based on the overall weight of evidence rather than on particular percentage(s). We have added the references again along the statement. We also clarified that the preceding citation (“Analysis of predictors of IQOS current use (use in the previous 30 days) in 2017…”) is linked to citation Tabuchi (2016) specifically.

 The Reviewer indicated that data related to the hotline and adverse events (AEs) should be provided in the manuscript. However, safety was not a primary or secondary objective of this study. AEs spontaneously reported by study participants were collected using a passive safety surveillance methodology. The safety population exposed to the product use included the 1,158 participants who used at least one *HeatStick* and their data were valid for safety analysis. Overall, 121 AEs (102 non-serious and 19 serious) were reported in 48 individual cases. The overall reporting rate was 4.14%. The most frequent reported AEs by preferred terms (PTs) were Headache (n=13), Malaise (n=7), Nausea (n=6), Dizziness (n=4), Abdominal discomfort (n=3), Oral discomfort (n=3), Chest pain (n=3), Cough (n=3), and Throat irritation (n=3). In total, the most frequent PTs represent 37.19% (45/121) of the total number of AEs reported during the study for all THS product variants. Headache was the most frequent reported AE in both product variants investigated in the study (reported in 13 out of 48 cases), with a reporting rate of 10.74% from all AEs received. Other frequent reported AEs were Malaise, Nausea and Dizziness (overall reporting rates from 3.30% to 5.78%). The severity of the leading event was reported as Mild in 5 cases (10.40%), Moderate in 7 cases (14.58%), Severe in 6 cases (12.50%), and Unknown in 6 cases (12.50%). The severity was not reported in half of the total number of cases (24 cases, 50.0%). Those details can be found as part of the full study report which is publicly available as part of Philip Morris Products S.A. Modified Risk Tobacco Product (MRTP) Applications and can be accessed through following link: https://www.fda.gov/tobacco-products/advertising-and-promotion/philip-morris-products-sa-modified-risk-tobacco-product-mrtp-applications.

 Regarding the study design and the chosen length of the observational period, the duration of six weeks was based on the assumption that participants would take between two and four weeks to either establish a regular pattern or to stop using *HeatSticks* altogether. The remaining weeks of the observational period would serve to confirm that *HeatSticks* usage patterns of participants have indeed stabilized. This study duration was also implemented by 22nd Century Group Inc. Modified Risk Tobacco Product (MRTP) Applications when conducting their actual use study pre-market. More information about 22nd Century Group Inc. Modified Risk Tobacco Product (MRTP) Applications can be accessed through the following link: https://www.fda.gov/tobacco-products/advertising-and-promotion/22nd-century-group-inc-modified-risk-tobacco-product-mrtp-applications.

 The Reviewer recommended to provide further details on the structure and validity of the questionnaires used, including which measure was implemented to capture participants’ intention to quit. The goal of our study was measure THS use patterns in current U.S. adult daily cigarette smokers and to assess THS product acceptance. Socio-demographics, smoking habits, sensory assessment, and ease of use were measured with ad-hoc questions. Consumption of cigarettes and THS were collected on self-reported stick-by-stick entry in an e-diary.

 To capture intention to quit smoking, participants were asked to complete the Prochaska Stages of Change measure. Using a dichotomous response scale (‘yes’, ‘no’), participants were first asked if they were seriously considering quitting smoking within the next six months. For those participants who indicated ‘yes’ for intention to quit smoking within the next six months, they were further asked if they were planning to quit smoking in the next 30 days. The Prochaska Stages of Change is based on the Prochaska and DiClemente stages of change concept, which is part of the Transtheoretical Model. The instrument assesses smokers’ mental state for intention to quit smoking and enables categorization of smokers into the following three stages: 1) precontemplation (not thinking of quitting smoking), 2) contemplation (thinking of quitting smoking within the next six months), and 3) preparation (thinking of quitting smoking within the next 30 days). Of note, the measure of intention to quit smoking within the next 30 days was applied for the corresponding exclusion criteria. This is because THS is aimed at current adult smokers who would otherwise continue to smoke combustible cigarettes.

 We also assessed intention to use THS as part of the inclusion criteria, however, measuring intention to switch to low-risk products in general was not performed. The full questionnaire (Case Report Form) can be found as part of the study report, which is publicly available as part of Philip Morris Products S.A. Modified Risk Tobacco Product (MRTP) Applications and can be accessed through the following link: https://www.fda.gov/tobacco-products/advertising-and-promotion/philip-morris-products-sa-modified-risk-tobacco-product-mrtp-applications.

 The Reviewer commented that the participants own cigarette brand could have been an important predictor for *IQOS* adoption. We did not collect the participants’ own cigarette brand as part of the survey, so we could not include this variable in the analysis.

 The Reviewer recommended to further discuss the results of the analyses for heavy smokers, i.e. the reduced adoption of THS among heavier smokers, and whether this may reflect high level of inefficiency of the product to adequately reproduce the experience in cigarette smoking. From our perspective, heavy adult smokers might simply need more time to adopt THS than current adult smokers who smoke fewer number of cigarettes per day.

# Peer review: Reviewer response for version 1

**DOI:** 10.5256/f1000research.19251.r51842

**Published:** 2019-08-28

**Authors:** Kenneth A. Mundt

**Affiliations:** 1 Cardno Chemrisk, Boston, MA, USA

**Summary**

 This article presents the methods and findings of a study in which a heated (not combusted) tobacco product (referred to as the Tobacco Heating System, or THS) was made available to current cigarette smokers who volunteered to participate and to maintain detailed diaries of their tobacco consumption, including cigarettes, THS, e-cigarettes, and any other nicotine-containing products. The study consisted of a one-week baseline period, six-weeks of observation, and one week ‘close-out’ period.

 Participants were invited from a large market research database. Approximately 1300 participants enrolled, of which 1100 met the minimal cigarette and THS use criteria (one each) and by the end of the study 965 had completed diaries and were included in the statistical analyses. In addition to data on tobacco product consumption, participants provided information on socio-demographics (sex, age, race/ethnicity, household information, marital status, educational attainment, occupation, income, etc.); smoking and tobacco use (number of cigarettes or THS or other products, intention to quit); and THS product assessment (taste, smell, aftertaste and ease of use).

 Patterns of tobacco product use and changes over the six-week observation period were reported. Logistic regression with stepwise inclusion/elimination of variables (and first order interaction terms) was used to identify predictors of THS adoption (defined as >70% THS use). Participants of older age, selecting both THS flavors, users of and more favorable product assessment (taste and ease of use factors) and those never having attempted to quit were significantly more likely to adopt THS. Gender (not statistically significant) and ethnicity (statistically significant) contributed less. 

 Study strengths and weaknesses are well presented. The main strength was that this study closely monitored actual use of cigarettes and THS (and other nicotine products) over six weeks. One unavoidable limitation is that THS was provided to participants at no charge, and whether the observed patterns would be different if THS users were required to purchase their supplies. 

**General comments**

 This study generated several interesting and potentially helpful insights regarding real-world selection and use of THS among current conventional cigarette smokers. It appears to be the first study to provide such preliminary insights regarding how THS might be received in the United States. 

 The study methods, data collected, statistical analyses and results all are clearly presented. However, the discussion remains somewhat thin, i.e., individual analytical findings are addressed in succession, but the overall conclusion and integrated findings have not been fully explored. Interpretations of study findings should be compared more broadly with the published literature on other non-combusted tobacco or nicotine-containing products, as well as the possible impact of THS adoption among current smokers in the US. While important insights were generated regarding the group of smokers who volunteered for the study, no inference can be drawn with regard to non-tobacco users or users of other nicotine-containing products. The need for post-marketing studies is noted, but suggestions for their objectives (or specific research questions) are not offered. What specific additional studies would build on the initial findings reported here?

 A more minor point: the text would benefit from careful editing to correct some grammatical errors and primarily to make it more idiomatic.

**Specific comments**

Abstract

 The abstract needs improvement in content as well as structure.

 Background: The final sentence presents the study aim; this largely is restated (with more detail) under the methods section. The reference to logistic regression reflects part of the study methods and should be moved.

 Methods: The only information provided is that tobacco users were observed over six weeks. A brief but more informative overview of the study design would be helpful.

 Results: This appears to be reasonably complete. Perhaps response rates (which were remarkably good) should be stated.

 Conclusions: This section largely repeats results (and only some) and recommends post-marketing studies, but provides no interpretations, synthesis or policy implications based on this study. 

Introduction

 The introduction is informative and well presented.

 Is it possible to provide some examples of MRTPs available in the US? Perhaps it would be helpful to clarify the status of e-cigarettes with respect to MRTPs.

Methods

 The methods are clearly described. A few suggestions:

 Setting: How or why were these study locations chosen? The reference to “Good Epidemiological Practice” should carry a citation, and perhaps be moved under Study Design.

 Products: THS is described in greater detail here, and does not reflect any methods. Perhaps the description of the three components of the THS should be moved to the introduction where the THS is first described.

 Data collection and measurements: There is no mention of what took place during the final “close-out” week (it may not belong here, however).

 Analysis: the logistic regression approach is reasonably clear, i.e., stepwise selection and (presumably) backward elimination to remove parameters with p<0.05. However, the choice of p<0.05 should be more clearly justified - what is the impact of eliminated parameters on the coefficients of those retained (i.e., is there evidence of confounding and does confounding increase when these terms are eliminated)?

Results

 Study participants: response/participation rates (which are remarkably good) should be presented.

 Potential predictors of adoption of THS: Adoption of THS by men and women differed by nearly 30% but was not statistically significant. Should this be presented as “no influence of sex”?

Discussion

 How might the statistically significant reduced adoption of THS among heavier smokers and among non-Latino smokers be interpreted? Did these groups increase their conventional cigarette use or simply fail to adopt THS as much as other groups? Might the results suggest that these smokers are more habituated or committed to smoking conventional cigarettes? 

 It seems intuitive that adoption of THS would be preferentially higher among those who found it easy to use and more enjoyable. Is there any alternative interpretation? Similarly, might study participants selecting both THS types reflect populations more interested in variety than single product loyalty? How might these observations be used to predict what might happen in the US if THS were broadly available to current conventional cigarette smokers? Can some quantitative range of projection(s) be made regarding what proportion of cigarette smokers might adopt THS, i.e., quit conventional cigarette smoking?

 I noticed that the two study locations in a US state (i.e., NC) where tobacco is an important crop – and cigarettes are produced – were the least likely to adopt THS. This is interesting and might be explored further.

 Japanese studies demonstrated higher rates of THS adoption among smokers intending to quit smoking combusted cigarettes. In contrast, this study demonstrated higher adoption of THS among those who never attempted to quit. How might this be explained? What are the characteristics of smokers not intending to quit but adopting THS (vs. those not adopting, or those intending to quit)?

Is the work clearly and accurately presented and does it cite the current literature?

Yes

If applicable, is the statistical analysis and its interpretation appropriate?

Yes

Are all the source data underlying the results available to ensure full reproducibility?

Yes

Is the study design appropriate and is the work technically sound?

Yes

Are the conclusions drawn adequately supported by the results?

Yes

Are sufficient details of methods and analysis provided to allow replication by others?

Yes

Reviewer Expertise:

Epidemiology with a primary focus on concepts and methods as used to evaluate occupational, environmental and consumer product exposures.

I confirm that I have read this submission and believe that I have an appropriate level of expertise to confirm that it is of an acceptable scientific standard, however I have significant reservations, as outlined above.

# Response

**Published:** 2021-06-20

**Authors:** Christelle Chrea

**Affiliations:** Philip Morris, Switzerland

**General comments**

- **Discussion – General comments**

Several parts of the Discussion section have been reworked to address the Reviewers comments on the Discussion.

 In particular, the Reviewer commented that, while important insights were generated regarding the group of adult smokers who volunteered for the study, no inference can be drawn with regard to non-tobacco users or users of other nicotine-containing products. We have reflected this comment in the Discussion as follows: “The sample was not representative of the U.S. current adult smoker population, which should be considered when interpreting the results.”

 Regarding the need for post-market studies, we outline in the Discussion that objectives of such research is to provide actual levels of THS adoption and use patterns once THS is commercially marketed in a given market (in the context of this manuscript, in the U.S.) and what are the drivers of adoption in the population.

**Specific comments**

- **Abstract**

The Abstract has been slightly edited according to the Reviewer’s comments on its content and structure, with no alteration to the message presented (i.e. removed repetitious sentences, as well as addition, restructuration and move of sentences to a different part of the abstract for clarity, etc…).

 As also recommended by the Reviewer, the overall text of the manuscript has been further edited to correct any grammatical errors and further improve the flow.

  

- **Introduction**

As suggested by the Reviewer, the Introduction was edited to present how other regulatory authorities are considering different types of products, including e-cigarettes, and to provide examples of the MRTPs available in the U.S. (including MRTP applications accepted for review).

 We also updated in the Introduction the status of the PMI MRTP Applications for *IQOS* to indicate that, in July 2020, the FDA authorized the following claim “AVAILABLE EVIDENCE TO DATE: The IQOS system heats tobacco but does not burn it. This significantly reduces the production of harmful and potentially harmful chemicals. Scientific studies have shown that switching completely from conventional cigarettes to the *IQOS* system significantly reduces your body’s exposure to harmful or potentially harmful chemicals.”, together with the appropriate citation. In addition, we specified that “As of March 31, 2021 *IQOS* was available in 66 countries.”

  

- **Methods**

The Methods section was edited to outline why the study locations were chosen (“The study locations were chosen to recruit a sufficiently large and diverse number of current U.S. adult daily smokers. In each city, C&C Market Research was operating a dedicated booth within a mall, which was used as study site.”).

 A citation has been added to reference “Good Epidemiological Practice” (Hoffmann, 2019), and this study characteristic remains indicated under Setting sub-section of the Methods, along with the information related to the Institutional Review Board.

 THS description has been moved under Introduction section as suggested.

 Regarding the final “close-out” week, the Data collection and measurements sub-section of the Methods has been edited as follows: “During the "close-out" week period participants were not required to record any data, however, they were able to call the toll-free telephone hotline for the continued surveillance of potential adverse events (AEs).”

 For the logistic regression described under Analysis sub-section of the Methods, we think that there is a misunderstanding. The backwards elimination was performed with a criteria p<0.05 as a selection threshold to retain the variable in the model, not to remove it. In addition, it was tested if the model could be improved using this procedure.

  

- **Results**

The Reviewer recommended to present the response/participation rates. The aim of this analysis was to assess the potential predictors of adoption of the Tobacco Heating System (THS) by current U.S. adult smokers after six weeks of THS product use. With this in mind, the response/participation rates are, therefore, of limited value. This being said, the response/participations rates can be found as part of the full study report, which is publicly available as part of Philip Morris Products S.A. Modified Risk Tobacco Product (MRTP) Applications and can be accessed through the following link: https://www.fda.gov/tobacco-products/advertising-and-promotion/philip-morris-products-sa-modified-risk-tobacco-product-mrtp-applications. 

 Regarding potential predictors of adoption of THS, the Reviewer suggested that, because our results showed that “Adoption of THS by men and women differed by nearly 30% but was not statistically significant,” it should be indicated as “no influence of sex”. We agree with this comment and we confirm that the Results section include the following wording: “No influence of sex was found” (after Figure 2).

  

- **Discussion – Specific comments**

The Reviewer requested how the statistically significant reduced adoption of THS among heavier smokers and among non-Latino smokers could be interpreted. Indeed, heavy current adult smokers might need more time to adopt THS than current adult smokers smoking fewer cigarettes per day. Actually, based on the literature, Latino smokers tend to consume fewer cigarettes per day than non-Latino smokers. Therefore, the results of non-Latino smokers in our sample are likely related to the findings on heavy smokers who have a reduced adoption of THS.

 The Reviewer asked whether some quantitative range of projection(s) could be made regarding what proportion of cigarette smokers might adopt THS. We think that a quantitative projection would go beyond the objective of the study and would be difficult to justify.

 The Reviewer noticed that the two study locations in a U.S. state (i.e., NC) where tobacco is an important crop – and cigarettes are produced – were the least likely to adopt THS, and suggested to further explore this aspect. The study locations (eight cities spread across the U.S.) were chosen to recruit a sufficiently large and diverse number of U.S. adult daily smokers. The study was aimed at assessing differences by demographic characteristics and smoking habits rather than by study location.

 The Reviewer asked our perspective on Japanese studies data, which reported higher rates of THS adoption among current adult smokers intending to quit smoking combusted cigarettes, compared to our study, which, in contrast, demonstrated higher adoption of THS among those who never attempted to quit. Comparing the data from Japan and the U.S. is difficult, because we would compare pre-market data (U.S. data) with post-market data (Japan data) and different cultural environment. When it comes to the characteristics of the current adult smokers not intending to quit but adopting THS, we would not expect a difference in profile.
